# Evaluation of esterification routes for long chain cellulose esters

**DOI:** 10.1016/j.heliyon.2019.e02898

**Published:** 2019-11-22

**Authors:** Pia Willberg-Keyriläinen, Jarmo Ropponen

**Affiliations:** VTT Technical Research Centre of Finland Ltd, Tietotie 4E, P.O Box 1000, FI-02044, VTT, Finland

**Keywords:** Materials chemistry, Cellulose, Esterification, Long chain cellulose ester

## Abstract

Long chain cellulose esters are internally plasticized bio-based materials, which have good future potential in several applications such as coatings, films and plastics. The long chain cellulose esters with different side chain lengths were synthesized using different esterification methods. When homogeneous esterification was used, the acyl chloride method was the most effective esterification method and cellulose esters prepared using this method have the highest degree of substitution values (DS). In this case, the long chain cellulose esters showed DS values from 0.3 to 1.3 depending on the side chain length of cellulose esters. CDI activation, vinyl transesterification and anhydride routes resulted in somewhat lower DS values. The cellulose was also pretreated with ozone, which decreased cellulose molar mass, and resulted in synthesized cellulose esters having higher DS and better reaction efficiency than untreated cellulose. When heterogeneous esterifications were used, only acyl chloride method seemed to work.

## 1 Introduction

Cellulose is a significant raw material for several industries, such as papers, textiles, foods, cosmetics and biomaterials. Furthermore, cellulose is one of the most common natural polymers and can thus be considered as renewable material (Edgar et al., 2001). One of the unique properties of cellulose is that it can be chemically modified (e.g. via esterification) to achieve a required function, for example thermoplasticity (Klemm et al., 2005).

Long chain cellulose esters are bio-based materials, which are synthesized from longer chain fatty acids (chain length of fatty substituent ≥ C6) than currently commercially available thermoplastic cellulose acetate (CA), cellulose acetate propionate (CAP) and cellulose acetate butyrate (CAB) esters (Havimo et al., 2011). These long chain cellulose esters also have relatively good mechanical and barrier properties and therefore show promising potential in several applications such as coatings, films and bio-plastics (Havimo et al., 2011; Cao et al., 2014; Willberg-Keyriläinen et al., 2018a; Willberg-Keyriläinen et al., 2018b).

Long chain cellulose esters can be produced using both homogeneous and heterogeneous methods. McCormick et al. (McCormick et al., 1985) reported that homogeneous cellulose esterification in LiCl/DMAc has many advantages compared to heterogeneous method. These advantages include e.g. more uniform distribution of the side chains along the backbone, better controllability of the total DS and also somewhat reduced consumption of reagents (McCormick et al., 1985; Nagel and Heinze, 2012; Sealey et al., 1996; Wu et al., 2004). Despite the advantages of the homogeneous method, in most cases, long chain cellulose esters are produced using heterogeneous conditions. The heterogeneous route is commonly chosen since there is still difficulties in recycling the expensive lithium salt used in homogeneous method (Ding et al., 2017; Edgar et al., 2001). However, in heterogeneous reactions, the resulting degree of substitution (DS) is usually lower due to poor accessibility of esterification reagents to the solid cellulose molecules (Wei et al., 2007).

Fatty acid chloride is powerful reagent to produce cellulose fatty acid esters (Malm et al., 1951). However, fatty acid chloride produces hydrochloric acid (HCl) as a by-product of the esterification reaction. HCl gas is corrosive, thus causing constraints in the reactor choices (Ding et al., 2017). Therefore it is important to limit cellulose degradation and neutralize formed HCl using base (e.g. pyridine, trimethylamine, DMAP (*N,N*-dimethyl-4-aminopyridine)) (Granström et al., 2008; Joly et al., 2005; Vaca-Garcia et al., 1998).

Fatty acids as such do not cause cellulose degradation, but they have very low reactivity towards cellulose hydroxyl groups (Vaca-Garcia et al., 1998). For that reason fatty acids must be changed into more reactive entities. For example *N*,*N′*-carbonyldiimidazole (CDI) has been reported as an activating agent for cellulose acylation (Hussain et al., 2004; Nagel and Heinze, 2012). In this CDI activation method, the acylation is promoted by an activated ester, which forms in situ from the corresponding carboxylic acid in the presence of CDI (Granström et al., 2008). This activation method allows mild reaction conditions and limited amounts of by-products (Hussain et al., 2004). The transesterification reaction with vinyl esters under mild reaction conditions has also been used in the synthesis of cellulose esters. Thus, vinyl esters instead of acyl chlorides can be used to reduce the use of hazardous substances and wastes (Cao et al., 2013; Ding et al., 2017; Jebrane et al., 2017; Wen et al., 2017). The leaving vinyl alcohol group immediately tautomerizes to acetaldehyde, which can then be easily removed from the reaction system due to its low boiling point (Brand et al., 2017; Jebrane et al., 2017).

In this study, the aim was to evaluate the different esterification routes for long chain cellulose esters and to find out the most effective route. The long chain cellulose esters with different side chain lengths (C8, C12 and C16) were synthesized using different homogeneous and heterogeneous esterification routes. Fatty acid chloride route, CDI activation route, vinyl transesterification route and anhydride route were tested. In this research, we have proven the influence of esterification route and cellulose side chain length on cellulose reactivity and on the degree of substitution. Also the effect of cellulose molar mass on the reaction efficiency was studied using two pulps with different molar mass.

## 2 Materials and methods

### 2.1 Materials

Vinyl esters (octanate, laurate, palmitate) and anhydrides (octanoic, lauric, palmitic) were purchased from TCI Europe. 1,1′-Carbonyldiimidazole (CDI) and 1,8-Diazabicyclo(5.4.0)undec-7-ene (DBU) were purchased from VWR Finland. All other commercial reagents were purchased from Sigma-Aldrich.

The cellulose in this research was commercial softwood dissolving pulp produced by Domsjö Fabriker AB, Sweden. Ozone treated pulp was prepared according to a method described by Willberg-Keyriläinen et al. (Willberg-Keyriläinen et al., 2016).

### 2.2 Esterification of cellulose with acyl chloride (FA-Cl)

The esterification of the cellulose with acyl chloride was performed using the method previously presented by Willberg-Keyriläinen et al. (Willberg-Keyriläinen et al., 2016; Willberg-Keyriläinen et al., 2017). In this homogeneous method, the cellulose was dissolved in LiCl/DMAc solution, and then, anhydrous pyridine (3.6 equivalents to cellulose anhydroglucose unit (AGU)) was mixed with the cellulose solution. In the heterogeneous method, cellulose and anhydrous pyridine were mixed together. After this, fatty acid chloride (octanoyl chloride (C8), lauroyl chloride (C12) or palmitoyl chloride (C16), 3.0 equivalents to cellulose AGU) was added dropwise to the cellulose mixture. The mixture was stirred for 16 h at 80 °C (homogeneous method) or 5h at 100 °C (heterogeneous method). The cellulose esters were precipitated and washed with ethanol. The cellulose laurate and cellulose palmitate esters were additionally washed with acetone.

### 2.3 Esterification of cellulose with CDI activated carboxylic acid (FA-CDI)

The esterification of the cellulose with CDI activated carboxylic acid was performed using a modified method presented by Nagel et al. (Nagel and Heinze, 2012). In the homogeneous method, cellulose was dissolved in LiCl/DMAc solution and in the heterogeneous method, cellulose was mixed with DMAc. *N*,*N′*-carbonyldiimidazole (CDI, 3.0 equivalents to cellulose AGU) was dissolved in DMAc at room temperature and then carboxylic acid (octanoic acid (C8), lauric acid (C12) or palmitic acid (C16), 3.0 equivalents to cellulose AGU) was added to CDI/DMAc mixture. The mixture was heated up to 60 °C and kept for 3 h and then added dropwise to the cellulose solution. This mixture was then stirred for 16 h at 80 °C (homogeneous method) or 5h at 100 °C (heterogeneous method). The cellulose esters were precipitated and washed with ethanol. The cellulose laurate and cellulose palmitate esters were additionally washed with acetone.

### 2.4 Esterification of cellulose using vinyl transesterification (FA-vinyl)

The esterification of the cellulose using vinyl transesterification was performed using a modified method presented by Ding et al. (2017). In the homogeneous method, cellulose was dissolved in LiCl/DMAc solution and in the heterogeneous method, cellulose was mixed with DMAc. Then the vinyl octanate (C8), vinyl laurate (C12) or vinyl palmitate (C16) (3 equivalents to cellulose AGU) and catalyst (DBU, 1,8-Diazabicyclo[5.4.0]undec-7-ene, 3 equivalents to cellulose AGU) was added dropwise to the cellulose mixture. The mixture was stirred for 1h at 50 °C. The cellulose esters were precipitated and washed with ethanol. The cellulose laurate and cellulose palmitate esters were additionally washed with acetone.

### 2.5 Esterification of cellulose with fatty acid anhydride (FA-Anhydride)

The esterification of the cellulose with fatty acid anhydride was performed using a modified method presented by Nawaz et al. (Nawaz et al., 2012). In the homogeneous method, cellulose was dissolved in LiCl/DMAc solution and in the heterogeneous method, cellulose was mixed with DMAc. Then, anhydrous pyridine (3.6 equivalents to cellulose anhydroglucose unit (AGU)) was mixed with the cellulose solution. After this, fatty acid anhydride (octanoic anhydride (C8), lauric anhydride (C12) or palmitic anhydride (C16), 3.0 equivalents to cellulose AGU) was added to the cellulose mixture. The mixture was stirred for 16 h at 80 °C (homogeneous method) or 5h at 100 °C (heterogeneous method). The cellulose esters were precipitated and washed with ethanol. The cellulose laurate and cellulose palmitate esters were additionally washed with acetone.

### 2.6 Size exclusion chromatography (SEC)

The molar mass distribution was defined by dissolving the pulp samples in 0.8% LiCl/DMAc eluent followed by size exclusion chromatography (SEC) measurements, using MiniMix columns equipped with a Waters 2414 Refractive Index Detector (Waters, USA). The molar mass distributions of pulps were calculated against Pullulan Standards.

### 2.7 Carbohydrate analysis of pulps

The carbohydrate content of pulps was analyzed according to NREL method (Hausalo, 1995; Sluiter et al., 2008). The resulting monosaccharides were quantified by HPAEC with pulse amperometric detection (Dionex ICS 3000 equipped with CarboPac PA1 column). The polysaccharide content in the samples was calculated from the corresponding monosaccharides using correction factors of 0.9 for hexoses and 0.88 for pentoses.

### 2.8 Nuclear magnetic resonance (NMR) spectroscopy

The solid state ^13^C CP/MAS NMR spectroscopy (ssNMR) analyses of cellulose esters were carried out using an Agilent 600 MHz NMR spectrometer (Agilent Technologies, USA) using 3.2 mm triple resonance magic angle spinning (MAS) probe head. All ssNMR experiments were carried out at 22 °C using MAS rate of 10 kHz, 10 000 scans and 10 s recycle time.

## 3 Results and discussion

### 3.1 Molar mass and carbohydrate composition of pulps

In this research, the effect of cellulose molar mass on the reaction efficiency of esterification reactions was studied. Two pulps with different molar masses were tested. The reference pulp (untreated pulp) was a commercial dissolving grade softwood pulp with molar mass 520 kDa. The same commercial pulp was then pretreated with ozone according to the method described in our previous article (Willberg-Keyriläinen et al., 2016) to reduce the molar mass to 84 kDa (treated pulp).

Carbohydrate composition was determined for both the untreated pulp and the treated pulp and the results are tabulated in Table 1. Glucose was the main monosaccharide in both pulps and its primary source was cellulose. Cellulose content was 84.2% and 79.1% for untreated pulp and for treated pulp, respectively. After ozone pretreatment, glucose, mannose and xylose contents were reduced, which indicate that ozone pretreatment affected relative the concentration of both cellulose and hemicellulose, when compared to the untreated pulp. This observation is well in line with our earlier results (Willberg-Keyriläinen et al., 2019).

**Table 1:** Carbohydrate composition of the untreated and treated pulps.

Composition∗	untreated pulp	treated pulp
Glucose	93.5 ± 1.1	87.9 ± 2.5
Mannose	1.6 ± 0.1	1.2 ± 0.0
Xylose	1.2 ± 0.1	0.9 ± 0.1
Arabinose	<0.1	<0.1
Galactose	<0.1	<0.1
Fructose	<0.1	<0.1
Rhamnose	<0.1	<0.1
Monosaccharides total (%)	96	90
Polysaccharides (%)	87	81

∗ mg monosaccharide/100mg dry pulp.

### 3.2 Preparation of long chain cellulose esters

**Fig. 1:**
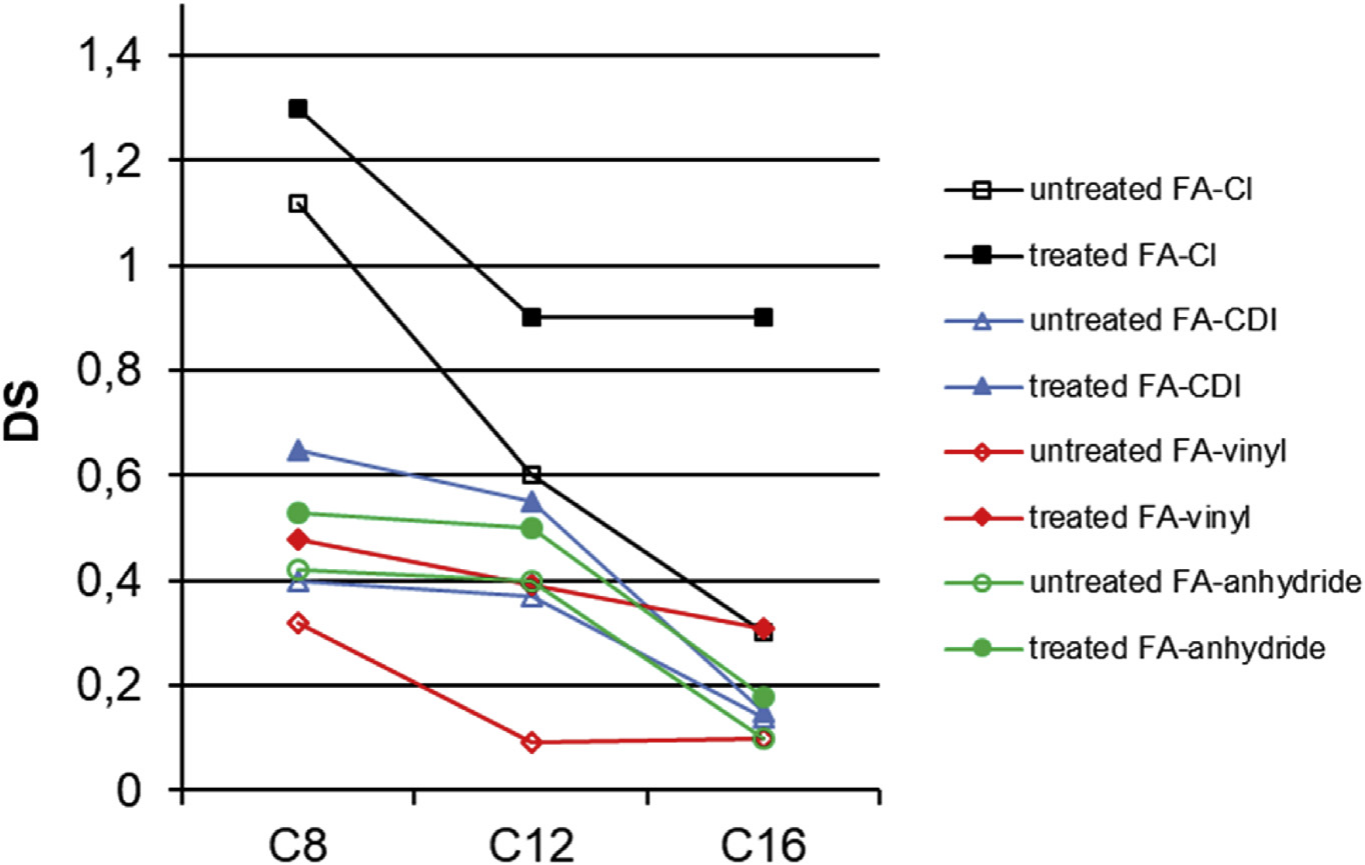
The degree of substitution for long chain cellulose esters prepared using different homogeneous esterification methods.

**Fig. 2:**
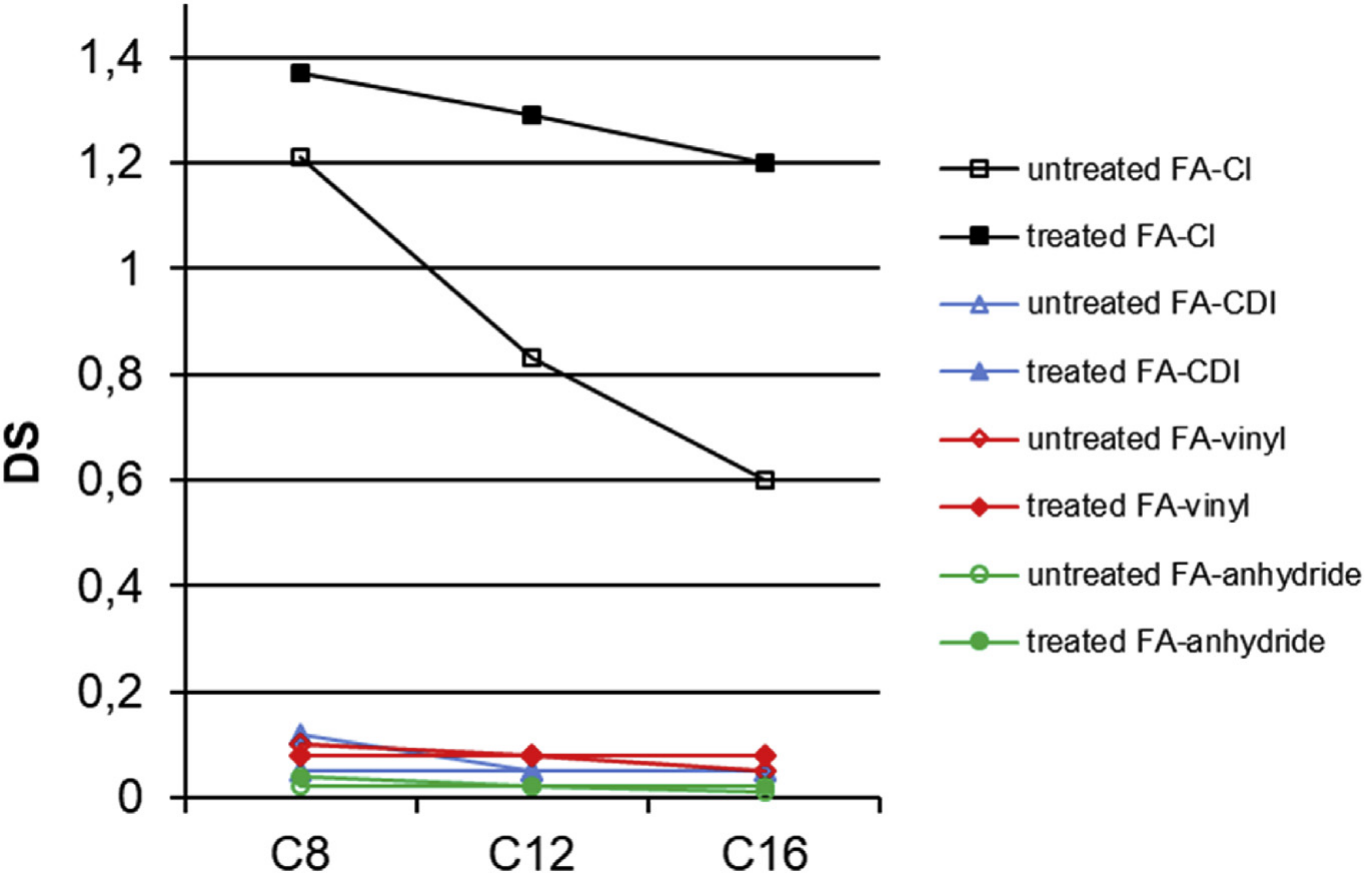
The degree of substitution for long chain cellulose esters prepared using different heterogeneous esterification methods.

To evaluate the effect of cellulose esterification method on the degree of substitution (DS), four different esterification methods were tested using constant equivalents of reagents. The esterification conditions (both homogeneous and heterogeneous) were chosen based on literature as such, and thus no additional optimization of conditions was done in order to have comparable results. DS values of long chain cellulose esters were analyzed using solid state NMR spectroscopy (ssNMR) by comparing the cellulose esters carbonyl carbon (173 ppm) integrals with the cellulose C1 signal (105 ppm) integral. The results are shown in Figs. 1 and 2.

The ssNMR was used, because all cellulose esters were not soluble with deuterated solvents and therefore liquid state ^1^H NMR measurement for characterizing the degree of substitution was not possible. King et al. (2010) have studied different NMR methods to determinate cellulose esters and they reported that in case of long chain cellulose esters the ^1^H NMR can suffer from poor resolution causing inaccuracies in DS determination. The ssNMR cross polarization (CP) experiment is capable of producing quantitative results, but this requires a careful calibration of the experimental parameters. In order to assure the quantitativity of the results the CP contact time was optimized so that all relevant nuclei had reached their maximum intensity, and no significant rotational frame relaxation had occurred. Hartmann-Hahn match for CP was calibrated using α-glycine, and verified with one of the studied samples. Finally, it was tested that the delay between successive scans was adequate to allow the protons to reach equilibrium magnetization before each scan.

Based on the DS values, we observed that when homogeneous esterification is used, the acyl chloride method is the most effective esterification method. In this case, cellulose esters showed DS values from 0.3 to 1.3 depending on the side chain length of cellulose esters. According to the results, it can be found that the DS values decrease significantly with increasing side chain length of cellulose esters. This is due to the steric hindrance of the aliphatic chain (Crépy et al., 2011; Freire et al., 2006; Willberg-Keyriläinen et al., 2017). Also the molar mass of the pulp can be said to have a significant impact on the DS values. The DS values of long chain cellulose esters from treated pulp are much higher than the values of esters from untreated pulp, while the untreated pulp has higher molar mass than treated pulp. This effect is the most significant when acyl chloride method is used and the side chain length of cellulose ester is longer. This is in line with our previous observation (Willberg-Keyriläinen et al., 2016), in which the ozone pretreatment decrease cellulose molar mass in controlled manner, enabling the synthesis of cellulose esters with significantly reduced amounts of chemicals than the synthesis of untreated pulp.

The DS values of cellulose esters prepared using homogeneous CDI activation route ranged from 0.7 to 0.1, which are lower than esters prepared using acyl chloride. In this method, the long chain fatty acids were activated in situ with CDI (N,N-carbonyldiimidazole), forming the imidazolides of fatty acid. The complete activation of fatty acid with CDI is necessary. The fatty acid imidazolide formation was monitored by ^13^C NMR with disappearance of fatty acid carbonyl shift at 176 ppm. We found that for long chain fatty acids (C12 and C16) the overnight activation with CDI at room temperature was not enough and the activation process was thus not complete. Some of the free fatty acids were left, which were not converted to the imidazolides of fatty acid. In case of octanoic acid (C8), activation was complete. When the activation was performed at 60 °C, 3 h was sufficient time to complete activation and convert all long chain fatty acids to imidazolide-complexes. However, it was observed that homogeneous esterification with fatty acid imidazolides was not efficient enough, and degrees of substitution were lower than for acyl chloride method. Possibly harsher conditions would be required to obtain higher DS values especially for longer chain lengths (C12 and C16).

The DS values of cellulose esters prepared using homogeneous transesterification with vinyl esters ranged from 0.5 to 0.1. Also in this method, the increase in fatty acid chain length had a negative impact on the DS. This is due to steric hindrance and decreasing solubility of vinyl esters in DMAc, when the length of aliphatic chain increases (Ding et al., 2017). Ding et al. (2017) have synthesized similar cellulose esters in LiCl/DMAc with vinyl esters as acylation agent. The DS of cellulose octanate (C8) ester was 1, when 6.5 eq/AGU of the vinyl octanate was used. They have concluded that the molar ratio of vinyl esters and DBU catalyst to anhydroglucose unit had a significant impact on the DS of cellulose esters. In our synthesis, we have used reagent 3eq/AGU in all experiments in order to have comparable results.

The DS values of cellulose esters, which were prepared using homogeneous esterification with anhydrides, ranged also from 0.5 to 0.1. Using anhydride method, the DS of cellulose octanate (C8) and cellulose laurate (C12) esters were quite the same. When the chain side length was increased to C16, the DS value dropped due to the steric effect.

When heterogeneous esterifications were used, only acyl chloride method seems to work. In this case, cellulose esters showed DS values ranged from 0.6 to 1.4 depending on the side chain length and molar mass of cellulose esters. When other esterification methods were used, the DS values were below 0.1. Using the heterogeneous acyl chloride method, the synthesized long chain cellulose esters showed somewhat higher DS values than with the homogeneous method. The effect was even more obvious with the untreated pulp, in which the DS values doubled from 0.3 to 0.6 with cellulose palmitate (C16). In case of cellulose octanate (C8), the difference in DS values is much smaller than for longer esters.

In the heterogeneous esterification reactions, the reaction solvent has also a great effect on successful preparation of cellulose esters. The solvent should not react with vinyl esters, long chain fatty acid imidazolides and anhydrides. Moreover, prepared cellulose esters should be also soluble in the used solvent (Cao et al., 2013). It seems that the prepared long chain cellulose esters are not completely soluble into DMAc solvent, which may limit the esterification reaction in heterogeneous conditions.

### 3.3 Solubility of long chain cellulose esters

The solubility of long chain cellulose esters into different solvents (acetone, DMSO, THF, toluene and chloroform) were tested for all cellulose esters prepared using homogeneous methods, and the results are shown in Table 2. Heterogeneous methods modified only cellulose fiber surface and the cellulose esters were not completely soluble in any of the solvents tested, even at a higher DS, due to the uneven distribution of the side chain groups. Fatty acids (C8, C12 and C16) as such were soluble in the tested solvents.

**Table 2:** Solubility of long chain cellulose esters in different solvents; esters prepared using the homogeneous esterification.

Cellulose	Esterification method	Side chain length	DS∗	Acetone	DMSO	THF	Toluene	CHCl_3_
treated	FA-Cl	C8	1.3	+	+	+	+	+
treated	FA-Cl	C12	0.9	-	+	+	+	+
treated	FA-Cl	C16	0.9	-	+	+	+	+
treated	FA-CDI	C8	0.7	-	-	-	+	-
treated	FA-CDI	C12	0.6	-	-	-	+	-
treated	FA-CDI	C16	0.2	-	-	-	-	-
treated	FA-vinyl	C8	0.5	-	-	-	-	-
treated	FA-vinyl	C12	0.4	-	-	-	-	-
treated	FA-vinyl	C16	0.3	-	-	-	-	-
treated	FA-anhydride	C8	0.5	-	-	-	-	-
treated	FA-anhydride	C12	0.5	-	-	-	-	-
treated	FA-anhydride	C16	0.2	-	-	-	-	-
untreated	FA-Cl	C8	1.1	+	+	+	+	+
untreated	FA-Cl	C12	0.6	-	-	-	+	-
untreated	FA-Cl	C16	0.3	-	-	-	-	-
untreated	FA-CDI	C8	0.4	-	-	-	-	-
untreated	FA-CDI	C12	0.4	-	-	-	-	-
untreated	FA-CDI	C16	0.1	-	-	-	-	-
untreated	FA-vinyl	C8	0.3	-	-	-	-	-
untreated	FA-vinyl	C12	0.1	-	-	-	-	-
untreated	FA-vinyl	C16	0.1	-	-	-	-	-
untreated	FA-anhydride	C8	0.4	-	-	-	-	-
untreated	FA-anhydride	C12	0.4	-	-	-	-	-
untreated	FA-anhydride	C16	0.1	-	-	-	-	-

soluble (+) and insoluble (-).

∗ According to ssNMR.

According to the solubility tests, it was found that cellulose esters with a DS > 0.9 were soluble in chloroform. This is complementing the results observed in our previous research (Willberg-Keyriläinen et al., 2016). The lower molecular weight of cellulose clearly has the advantage of the producing long chain fatty acid cellulose esters with higher DS. This also produces more plasticized cellulose material, which may also contribute to better solubility. Therefore these esters were soluble in chloroform with clearly lower DS values than earlier reported (Crépy et al., 2009; Heinze et al., 2003; Joly et al., 2005).

Cellulose octanate esters with DS 1.1 and 1.3 were soluble in all tested organic solvents. These cellulose octanate esters were the only ones, which were soluble in acetone. Cellulose esters with DS more than 0.6 were also soluble in toluene. The cellulose esters from untreated cellulose have lower DS values than cellulose esters from ozone treated cellulose, and therefore have poorer solubility into solvents.

Nagel et al. (Nagel and Heinze, 2012) and Ding et al. (2017) have also studied the solubility of cellulose esters in different solvents. They have concluded that solubility of cellulose esters decreases when side chain length increases, but they have also reported the solubility of cellulose esters being greatly proportional to the DS value. Based on our results the conclusion is similar - it is difficult to determine whether the side chain length or the DS values have a greater effect on the solubility.

## 4 Conclusions

We have shown that long chain cellulose esters with different side chain lengths can be synthesized using different esterification methods. When homogeneous esterification was used, the acyl chloride method was the most effective esterification method and long chain cellulose esters prepared using this method have the highest degree of substitution values (DS). In this case, the DS values ranged from 0.3 to 1.3 depending on the cellulose esters side chain length. CDI activation, vinyl transesterification and anhydride routes resulted somewhat lower DS values. When cellulose pulp was pretreated with ozone, the molar mass of cellulose decreased, and resulted in synthesized cellulose esters having higher DS and better reaction efficiency than untreated cellulose. When heterogeneous esterifications were used, only acyl chloride method seems to work. The solubility of long chain cellulose esters was mainly dependent on the degree of substitutions; the higher DS led to better solubility of esters with all fatty acid chain lengths.

## Declarations

### Author contribution statement

Pia Willberg-Keyriläinen: Conceived and designed the experiments; Performed the experiments; Analyzed and interpreted the data; Contributed reagents, materials, analysis tools or data; Wrote the paper.

Jarmo Ropponen: Conceived and designed the experiments; Analyzed and interpreted the data; Wrote the paper.

### Funding statement

This research did not receive any specific grant from funding agencies in the public, commercial, or not-for-profit sectors.

### Competing interest statement

The authors declare no conflict of interest.

### Additional information

No additional information is available for this paper.
